# Identifying community physical activity and health resources for treatment of back pain by utilizing members of a physical activity network

**DOI:** 10.1186/s12889-020-09569-6

**Published:** 2020-10-01

**Authors:** Cara L. Sedney, Treah Haggerty, Samuel Zizzi, Patricia Dekeseredy

**Affiliations:** 1 School of Medicine, West Virginia University, Morgantown, USA; 2 College of Physical Activity and Sport Sciences, West Virginia University, Morgantown, USA; 3 WVU Medicine, Morgantown, USA

**Keywords:** Physical activity, Capacity building, Rural health, Collaborative research

## Abstract

**Background:**

Back pain is one of the leading causes of health care expenditure in the US and is linked to an increased body mass index. Many evidence-based modalities for the prevention and treatment of back pain closely mirror recommendations for weight loss and include physical activity and health maintenance activities (PAHM). The primary aim of this study was to ascertain community assets, and perceptions of the use of PAHM in the treatment of back pain by West Virginia Physical Activity Network (WVPAN) members.

**Methods:**

Participants for the study were recruited from the West Virginia Physical Activity Network. This grassroots organization is filled with volunteers from various sectors who were recruited from various workshops, conferences, or coalition meetings over a period of several years. This network was purposely selected as the study population because of the statewide reach and their familiarity with resources in their local communities. A brief survey instrument was designed to gather their scaled perceptions about various treatment modalities related to back pain, and to gather their local knowledge related to specific providers in their communities. In addition, participants were given a free text box to list any local assets or resources for the nine treatments listed, and county of residence, and the nature of their connection to the physical activity network. Descriptive analyses were used to describe overall patterns of survey data. The qualitative data were compiled manually by the research team to show themes of specific treatments mentioned across different parts of the state.

**Results:**

Participants overwhelmingly supported physical therapy, flexibility training, yoga, and core strengthening as treatments for back pain. The majority of respondents were “undecided” about other treatments such as cognitive behavioral therapies and acupuncture.

**Conclusions:**

The implementation of PAHM interventions in communities could help treat patients with back pain, and may reduce reliance on the pharmacological treatment for back pain. The current study’s data support the potential of such approaches in many West Virginia counties. Also, local resources, and context can be gleaned from community leader surveys utilizing previously developed infrastructure for PAHM promotion.

## Background

Back pain is one of the leading causes of health care expenditure in the US and is linked to an increased body mass index (BMI) [1]. The prevalence of obesity is increasing in the nation (Centers for Disease Control [CDC], 2018) [2]. Many evidence-based modalities for the treatment of back pain closely mirror recommendations for weight loss [3]. These recommendations include physical activity and health maintenance activities (PAHM). In addition to helping people be more active, health maintenance activities are linked to preservation of health. The implementation of PAHM interventions has been problematic nationwide in spite of the strong evidence in favor of such interventions [4–6]. Barriers to implementation noted by these researchers include location, lack of time, lack of motivation, inconvenience, and lack of support [4–6]. Systematic studies regarding the viability and implementation of such interventions in high risk communities are warranted.

West Virginia leads the nation in rates of adult with obesity as well as obesity-related chronic diseases such as type 2 diabetes and hypertension [7]. Another, non-silent complication of obesity is back pain, which has a social and economic significance in its own right. The implementation of PAHM interventions in communities could help treat patients with back pain, and may reduce reliance on the pharmacological treatment for back pain.

There is a growing body of evidence that community based efforts can be effective in promoting physical activity [8–10]. Programs that include personal contact and are tailored to the specific characteristics of the community have proven to be most effective [11]. These interventions take on additional importance in rural environments where social and cultural factors may affect sustainability. Fortunately, many individuals and groups are passionate about improving physical activity and healthy lifestyles in their local communities. In West Virginia, one such organization is the West Virginia Physical Activity Network (WVPAN). The WVPAN aims to increase physical activity by assisting communities to create a culture of a physical activity [12]. The majority of members included in the physical activity network email list provided their contact information at various workshops, conferences, or coalition meetings over a period of several years. Most of the members have professional ties to the network such as a formal role in public health or as a physical educator in the school system. Members of the network serve as “community leaders” or” physical activity activists” to promote and assist in local efforts to improve health through the culture of physical activity. The interventions within WVPAN consist of many options including walking groups and biking clubs along with competitive running (e.g., trail races, community 5 k). This organization also maintains a directory of physical activity groups around the state, supports individuals initiating physical activity, helps organize events to promote physical activity, and share stories of what’s happening in the state around physical activity. The WVPAN also lists other partners for physical activity and health on its website [12].

This pilot study is the first step in planned implementation of evidence-based interventions to promote physical activity as recommended by Rabin and colleagues [13]. Rabin’s suggestions include conducting a prestudy to ensure recommendations build upon individual and organizational capacity. This information is meant to inform trial interventions at primary care facilities in several counties where viable and available PAHM resources were identified through an additional partnership, with the Practice Based Research Network (PBRN). The region-specific results of the pilot study will support primary health care providers in considering non-pharmacological treatment interventions for patients with back pain. Thus, the primary purpose of the study was descriptive in nature, specifically to inform future intervention work in specific WV communities. The aim of the current study was to collaborate with WVPAN leadership, specifically SZ (Sam Zizzi, coauthor of this paper), to ascertain the assets (resources available in their community) and context (circumstances that impact the acceptability and accessibility of physical activity resources) of the use of PAHM in the treatment of back pain by WVPAN members as community leaders.

## Methods

### Sampling and recruitment

Participants for the study were recruited from the WVPAN. This grassroots organization is filled with volunteers from various sectors (e.g., education, business, healthcare, and fitness) who have signed up via various workshops, conferences, or coalition meetings. Members of the organization come from all areas of the state, and there is a mix of both professionals and community lay leaders. Communications that go out to the WVPAN are managed by the Center for ActiveWV within the College of Physical Activity and Sport Sciences at West Virginia University. The network was purposely selected as the study population because of the statewide reach and their familiarity with resources in their local communities.

### Survey instrument

The brief survey instrument was designed to gather their scaled perceptions about various treatment modalities related to back pain, and to gather their local knowledge related to specific providers in their communities. Thus, the primary dependent variables were the respondents’ perceptions of the use of nine treatments for back pain. For these scaled items, the members were asked to indicate their agreement or disagreement using a five-point Likert type scale with nine PAHM treatments for back pain. The scale varied from “Strongly Disagree” (1) to “Strongly Agree” (5). These modalities included physical therapy, chiropractic care, aerobic exercise, flexibility training, massage, acupuncture, yoga/Pilates/tai-chi, medication use (i.e., NSAIDs), and cognitive behavioral therapies. A free text box was included after the scaled items asking participants to list any local assets or resources for the nine treatments listed. County of residence and the nature of their connection to the physical activity network (e.g., healthcare provider, fitness provider, government organization, educator, etc.) were also collected. No additional demographic variables about participants were collected.

### Procedures

After Institutional Review Board approval, an online survey was emailed to all 1248 members of the WVPAN using the online survey system, REDCap. REDCap is a secure web-based application utilized by West Virginia University [14]. A week later, a reminder email was sent to members who had not responded to the survey. Additionally, a live link to the questionnaire was also posted on the WVPAN member Facebook page. An IRB approved cover letter was included in the email sent to the members to describe the study. Participants indicated their written consent to participate by checking a box that would then open the survey. All returned questionnaires were received anonymously through the REDCap application.

### Statistical analyses

Since the primary purpose of the study was exploratory, descriptive analyses were used to describe overall patterns of results for the scaled data. Specifically, means and standard deviations were generated for the scaled items and percentages were calculated for the categorical items. These quantitative analyses were conducted directly within the REDCap program. For the open text responses, qualitative content analysis was applied to the data using an inductive approach. The most common responses were compiled manually by the research team to show themes of specific treatments mentioned across different parts of the state.

## Results

After two email reminders, 145 responses to the email survey were received covering 41 of the 55 (73%) counties in WV, resulting in an 11.6% response rate. The most members were from two of the higher populated areas of West Virginia, Kanawha (16%) and Monongalia counties (17%). Five members who completed a survey did not indicate their county of residence.

The WVPAN membership includes formal and informal connections to physical activity. Some are employees or owners of fitness related businesses. Others are teachers or health care providers. We asked the respondents to self-identify with one of six categories. Fitness enthusiast was included as a category to capture those with no formal or professional role in physical fitness but have joined the WVPAN to stay connected with the networks’ activity. Approximately 75% of respondents were associated with the WVPAN in a formal role through their work as an educator, government employee, fitness instructor, or healthcare provider. The greatest number of respondents indicated their affiliation with the WVPAN was as an educator (43%). Seven respondents did not indicate their affiliation. The details of the members’ connection to the WVPAN are presented in Table 1.

**Table 1:** Respondent’s self-identified affiliation to WV Physical Activity Network

Affiliation	Percent of respondents
Educator	43%
Fitness enthusiast^a^	24%
Belongs to a government agency	17%
Healthcare provider	7%
Own a health related business	5%
Fitness instructor	4%

*N* = 138

^a^Fitness enthusiast defined as those with no formal role within the WVPAN or in their community

**Fig. 1:**
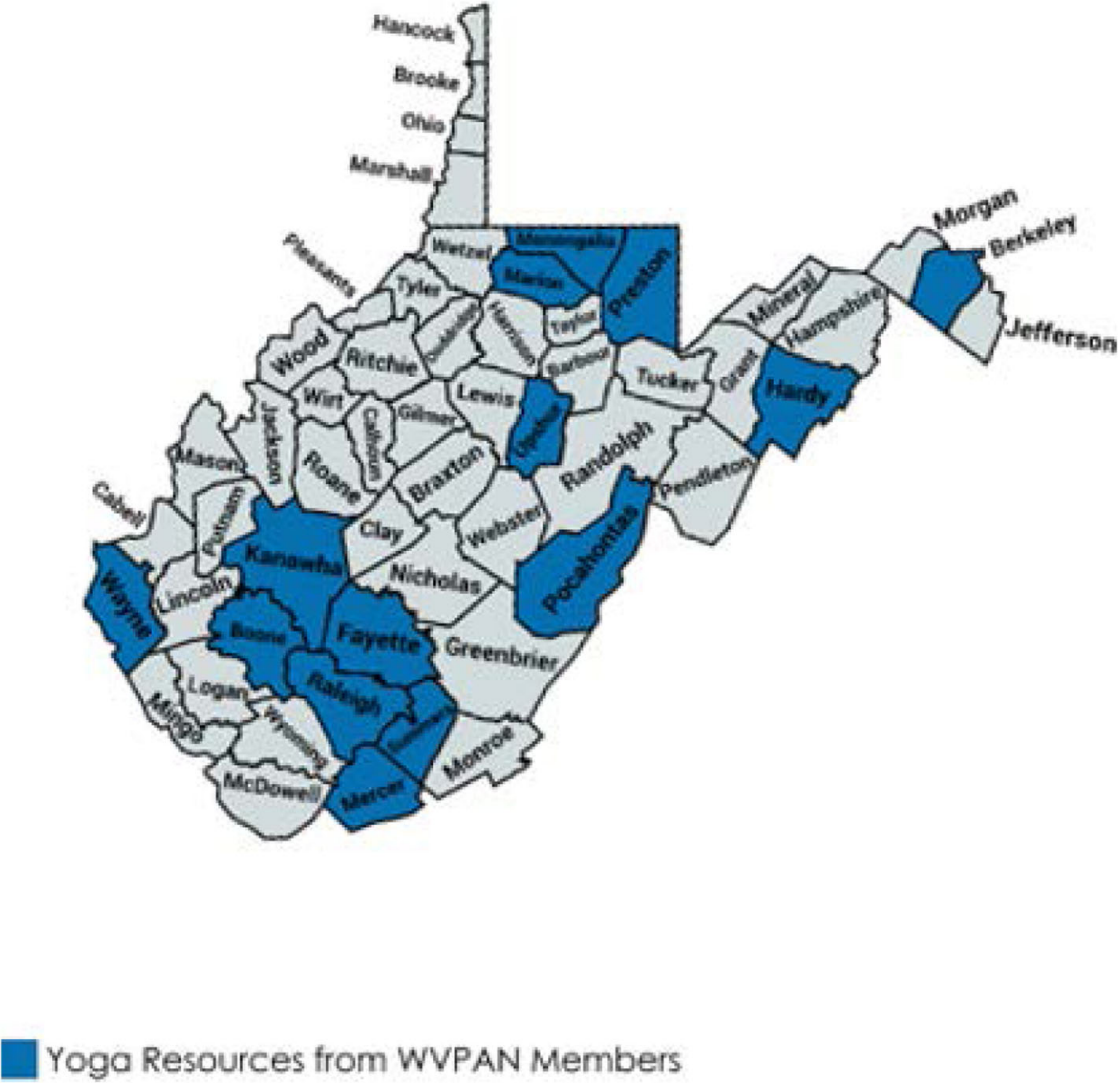
Yoga resources from WVPAN members. *Counties with at least one yoga resource identified by WVPAN member.* Map created with the permission of mapchart.net

Members of the WVPAN overwhelmingly agreed that physical therapy is important for back pain with 93% indicating they agreed or strongly agreed with this statement. Other physical activities such as flexibility training and core strengthening were also highly supported at 95%. The majority of respondents were “undecided” about other treatments such as cognitive behavioral therapies and acupuncture. Details of survey responses are represented in Table 2. The WVPAN members were asked to identify local resources or assets for PAHM treatments for back pain that they felt might be of interest to patients or health care providers. Free-text answers included dance, swimming, stress reduction, and general wellness. The most suggestions were again from the members in the most populous counties in the state. The most referenced resource was yoga, which was well represented across the state in 15 counties. Counties where yoga was referenced as a resource for treating low back pain are indicated in Fig. 1.

**Table 2:** Responses to survey questions about non-opiate back pain^a^

Survey question	Agree/strongly agree	Undecided	Disagree/strongly disagree
Flexibility training is important in the treatment of back pain (*N* = 141)	96%	3%	1%
Aerobic exercise and core strengthening are important in the treatment of back pain (*N* = 145)	95%	3%	2%
Physical therapy is important for the treatment of back pain (N = 145)	93%	5%	2%
Massage is important in the treatment of back pain (N = 145)	81%	15%	4%
Yoga/Pilates/Tai Chi are important in the treatment of back pain (N = 145)	71%	26%	3%
Chiropractic care/ spinal manipulation is important in the treatment of back pain (*N* = 144)	66%	26%	8%
NSAIDS are important in the treatment of back pain (N = 141)	52%	37%	11%
Cognitive/Behavioral therapies are important in the treatment of back pain (*N* = 142)	46%	48%	6%
Acupuncture is important in the treatment of back pain (N = 145)	38%	54%	8%

^a^rounded to whole number

Along with the business names of local resources, people also suggested therapists and instructors by name giving them personal recommendation. Some participants referenced a lack of affordable alternative treatments for back pain. For example: *“*Here in lies the problem; no facilities of note here in Monroe County other than physical therapists. We need to make AFFORDABLE and practical alternatives available here in Monroe County”. “There are very few resources in my small town. Sporadic yoga classes at the Stockert Youth Center. Massage therapy is very limited.”

Another participant added:

In the Newell, Chester, and New Cumberland area, there are very little opportunities for people seeking any type of exercise and core strengthening programs for our citizens. Schools have zero funds for physical education programs other than the annual faculty senate funds. I feel our community does not find physical fitness important in their lives :( .

Others described the challenges accessing resources that could be available:

In the southern part of WV access to some of the above treatment methods are not available locally. Sometimes you have to drive an hour or more to get those treatments. Sometimes insurance companies do not cover the treatment. For example my insurance through my job only covers 20 chiropractic care visits and mental health is limited and if it is approved you have a higher deductible which you cannot afford. My insurance also doesn't cover any gym membership, we are in a work crisis in the southern part of the state with the industries. We can barely afford to put food on the table much less purchase a gym membership.

## Discussion

In this study, we sought to assess community resources, assets, and context for the utilization of PAHM for treatment of back pain through a previously developed community network. Within the Consolidated Framework for Implementation Research Constructs, the nature and quality of formal and informal networks, basic assumptions, attitudes, and shared perception of importance amongst stakeholders, as well as available resources must be assessed and accounted for [15]. In addition, “overcoming indifference or resistance” amongst “champions,” or community leaders, is a key component of an implementation intervention.

Previous work by our group has demonstrated an openness amongst patients with back pain in a subspecialist clinical setting to discuss PAHM and other weight loss strategies as a way to aid in the treatment of back pain, but that implementation of PAHM is challenging without specific regional knowledge of local PAHM resources [16]. This survey of WVPAN community leaders gives an overview of possible PAHM resources throughout the state while also assessing attitudes towards PAHM for the treatment in back pain amongst this group of stakeholders. Importantly, PAHM resources appear to be available throughout the state, although possibly not in every county. More unique physical activity resources were identified in the counties that had more members responding to the survey while yoga was more widespread throughout the state. The survey also assesses the potential buy-in of future implementation interventions by community leaders, whom seem to strongly support PAHM methods as a treatment for back pain. In particular, physical therapy, yoga and flexibility training, and aerobic exercise were felt to be of benefit. These modalities may be areas of particular interest for further study.

This study contributes to the growing body of community engaged research. This process involves “working collaborative with groups of people affiliated by geographic proximity, special interest, or similar situations with respect to issue affecting their well-being” [17]. In this study, we partnered with the WVPAN, a group of people who share a special interest in physical activity in West Virginia. Community engagement is a proven strategy to identify priorities for physical activity and healthy lifestyles in unique communities. For example, Fialkowski et al., engaged with the Children’s Healthy Living Program to identify environmental priorities to address childhood obesity in remote and underserved populations in the USA and affiliated Pacific Islands. Researchers found the community leaders and local physical activity activists and those invested in childhood health were grateful for the opportunity to discuss childhood obesity and provide input [18]. One of the priorities discussed was promoting physical activity.

Community involvement is essential when seeking local knowledge and context [19]. Integrating community-based knowledge in research, in turn benefits the community. Moreover, members who provide local knowledge informed by their experiences contribute to greater program success and sustainability [19]. By engaging with the WVPAN to identify PAHM to treat back pain, we found many locally identified resources to inform future interventions aimed at providing acceptable options for treatment. These results are similar to John et al., who engaged community members to create an attribute map though photos exploring local resources for diet and physical activity [20]. In addition, we were also able to glean information from the local residents that we would not be able to find by an internet search. Participants gave personal recommendations by name and some expressed a complete lack of resources altogether. The current study demonstrates the feasibility of partnering with grassroots organizations such as the WVPAN for a novel approach of engaging members of a state wide community, as partners in research.

Limitations of this study include bias as a result of its survey methodology, as well as response rate, and single state location. This method was purposefully selected, however, to fit the main aim of the study. Almost half of the respondents identified as educators followed by fitness enthusiasts at 24% leaving little representation from the other four groups. Some participants wrote that there was an overall lack of resources in their communities but we do not know if the others left this section blank because of a lack of resources or simply by choice. Because community leaders with an established interest in PAHM were surveyed, it is not known if buy-in would be similar amongst others in a rural West Virginia community. Survey respondents with strong feelings regarding PAHM may have been more likely to follow through with the survey, which may have biased results towards more favorable perception of PAHM. This bias is likely magnified by the low response rate of 11.6%, which nevertheless may be somewhat mitigated by the sample size. However, in future work in these communities, this bias towards the implementation of PAHM may benefit researchers and clinicians as participant buy-in could already by established, allowing a vehicle for intervention. Also, the participants were from a single, mostly rural state. This may limit generalizability to other areas or populations.

## Conclusion

This pilot study was the first step in planned implementation of evidence-based interventions to promote physical activity as a treatment for back pain. Local resources and context can be gleaned from community leader surveys utilizing previously developed infrastructure for PAHM promotion. However, with a low response rate to the survey, further work is needed as these results just represent the attitudes of this group. Nevertheless, this preliminary data can provide valuable input for the construction of intervention development regarding utilization of PAHM in rural West Virginia community settings.

## Data Availability

All data generated or analyzed during this study are included in this published article.

## Abbreviations

BMI: Body mass index
PAHM: Physical activity and health maintenance activities
WVPAN: West Virginia Physical Activity Network

## References

[1] Dieleman J, Baral R, Birger M, Bui A, Bulchis A, Chapin A (2016). US spending on personal health care and public health, 1996-2013. JAMA..

[2] Adult Obesity Facts | Overweight & Obesity | CDC. Cdc.gov. 2018 [cited 2018 Oct 31]. Available from: https://www.cdc.gov/obesity/data/adult.html.

[3] Jensen M, Ryan D, Apovian C, Ard J, Donato K, Hu F (2013). AHA/ACC/TOS guideline for the Management of Overweight and Obesity in adults. J Am Coll Cardiol.

[4] Overcoming barriers to physical activity. Cdc.gov. 2017 [cited 2018 Oct 31]. Available from: https://www.cdc.gov/physicalactivity/basics/adding-pa/barriers.html.

[5] Guthold R, Stevens G, Riley L, Bull F (2018). Worldwide trends in insufficient physical activity from 2001 to 2016: a pooled analysis of 358 population-based surveys with 1·9 million participants. Lancet Glob Health.

[6] Sallis JF, Hovell MF (1990). Determinants of exercise behavior. Exercise and Sport Science Reviews.

[7] State Briefs [Internet]. The State of Obesity. 2019 [cited 2018 Oct 31]. Available from: https://stateofobesity.org/states/wv/.

[8] Abildso CG, Zizzi SJ, Reger-Nash B (2010). Evaluating an insurance-sponsored weight management program with the RE-AIM model, West Virginia, 2004-2008. Prev Chronic Dis.

[9] Bock C, Jarczok MN, Litaker D (2014). Community-based efforts to promote physical activity: a systematic review of interventions considering mode of delivery, study quality and population subgroups. J Sci Med Sport.

[10] Moore M, Warburton J, O’Halloran P, Shields N, Kingsley M (2015). Effective community-based physical activity interventions for older adults living in rural and regional areas: a systematic review. J Aging Phys Act.

[11] Strategies to prevent obesity and other chronic diseases: The CDC guide to strategies to increase physical activity in the community [Internet]. Cdc.gov. 2011 [cited 2018 Oct 6]. Available from: http://www.cdc.gov/obesity.

[12] About the West Virginia Physical Activity Network [Internet]. Facebook. [cited 2018 Oct 31]. Available from: https://www.facebook.com/.

[13] Rabin B, Brownson R, Kerner J, Glasgow R (2006). Methodologic challenges in disseminating evidence-based interventions to promote physical activity. Am J Prev Med.

[14] Harris P, Taylor R, Thielke R, Payne J, Gonzalez N, Conde J (2009). Research electronic data capture (REDCap)—a metadata-driven methodology and workflow process for providing translational research informatics support. J Biomed Inform.

[15] Damschroder L, Aron D, Keith R, Kirsh S, Alexander J, Lowery J (2009). Fostering implementation of health services research findings into practice: a consolidated framework for advancing implementation science. Implement Sci..

[16] Sedney C, Haggerty T, Dekeseredy P (2018). A short weight loss intervention in a neurosurgical subspecialist clinical setting. J Neurosci Rural Pract.

[17] Clinical and Translational Science Awards Consortium (2011). Community engagement key function committee task force on the principles of community engagement. Principles of community engagement.

[18] Fialkowski MK, DeBaryshe B, Bersamin A, Nigg C, Guerrero RL, Rojas G (2014). A community engagement process identifies environmental priorities to prevent early childhood obesity: the children’s healthy living (CHL) program for remote underserved populations in the US affiliated pacific islands, Hawaii and Alaska. Matern Child Health J.

[19] Glanz K, Rimer BK, Viswanath K. Health behavior: theory, research, and practice: San Francisco: Wiley; 2015.

[20] John D, Winfield T, Etuk L, Hystad P, Langellotto G, Manore M (2017). Community-engaged attribute mapping: exploring resources and readiness to change the rural context for obesity prevention. Prog Community Health Partnersh.

